# GloRESatE: A dataset for global rainfall erosivity derived from multi-source data

**DOI:** 10.1038/s41597-024-03756-5

**Published:** 2024-08-27

**Authors:** Subhankar Das, Manoj Kumar Jain, Vivek Gupta, Ryan P. McGehee, Shuiqing Yin, Carlos Rogerio de Mello, Mahmood Azari, Pasquale Borrelli, Panos Panagos

**Affiliations:** 1 Department of Hydrology, Indian Institute of Technology Roorkee, Roorkee, India; 2 School of Civil and Environmental Engineering, Indian Institute of Technology Mandi, Mandi, India; 3 Agricultural and Biosystems Engineering, Iowa State University, Ames, Iowa, USA; 4 Faculty of Geographical Science, Beijing Normal University, Beijing, China; 5 Water Resources Department, Federal University of Lavras, Lavras, Brazil; 6 Department of Range and Watershed Management, Ferdowsi University of Mashhad, Mashhad, Iran; 7 Department of Science, Roma Tre University, Rome, Italy; 8 Department of Environmental Sciences, Environmental Geosciences, University of Basel, Basel, Switzerland; 9 European Commission, Joint Research Centre (JRC), Ispra, Italy

**Keywords:** Hydrology, Natural hazards, Environmental impact

## Abstract

Numerous hydrological applications, such as soil erosion estimation, water resource management, and rain driven damage assessment, demand accurate and reliable rainfall erosivity data. However, the scarcity of gauge rainfall records and the inherent uncertainty in satellite and reanalysis-based rainfall datasets limit rainfall erosivity assessment globally. Here, we present a new global rainfall erosivity dataset (0.1° × 0.1° spatial resolution) integrating satellite (CMORPH and IMERG) and reanalysis (ERA5-Land) derived rainfall erosivity estimates with gauge rainfall erosivity observations collected from approximately 6,200 locations across the globe. We used a machine learning-based Gaussian Process Regression (GPR) model to assimilate multi-source rainfall erosivity estimates alongside geoclimatic covariates to prepare a unified high-resolution mean annual rainfall erosivity product. It has been shown that the proposed rainfall erosivity product performs well during cross-validation with gauge records and inter-comparison with the existing global rainfall erosivity datasets. Furthermore, this dataset offers a new global rainfall erosivity perspective, addressing the limitations of existing datasets and facilitating large-scale hydrological modelling and soil erosion assessments.

## Background & Summary

Land degradation is a growing global threat to ecosystem goods and services^1^. It is a human-induced phenomenon that reduces the capacity of soil to support life. As the world population continues to increase, the pressure on soil resources increases, and food security declines^2^. Soil erosion is a leading process of land degradation due to the detachment of fertile topsoil layers, which plays a vital role in global food security, water security, ecosystem services, and climate change abatement^3,4,5^. It was estimated that human actions may be responsible for nearly 60% of soil erosion^6^. Water and wind erosion detach 75 billion metric tons of soil annually, with agricultural land accounting for most of these losses^7^. The most important type of water erosion is topsoil erosion (splash and sheet processes), which is estimated to occur on 920 M ha of global land, while rill and gully erosion occur on 175 M ha of global land^8^. According to the report of United Nations^9^, erosion is one of the significant threats to the soil and impedes progress towards achieving the Sustainable Development Goals (SDGs) related to SGD15 (Life on land) and SGD2 (Zero hunger).

Rainfall erosivity (R-factor) is one of the critical drivers of the soil erosion processes with the greatest spatiotemporal variability^10,11^. Rainfall erosivity is generally estimated from empirical relations using storm kinetic energy and maximum 30-min rainfall intensity utilising pluviographs of more than 20 years^12^. In the early 21^st^ century, Yang *et al*.^6^ estimated global-scale rainfall erosivity and soil erosion change due to climate and land use changes using the Revised Universal Soil Loss Equation (RUSLE). Further, the Global Land Degradation Information System (GLADIS)^13^ database was also prepared to provide insight into the global land degradation status and trends. A lack of relatively high temporal-resolution (1–60 min) global rainfall datasets forced these pioneering studies to use medium-resolution (monthly and annual) rainfall datasets for rainfall erosivity estimation. In recent periods, Naipal *et al*.^14^ improved the global rainfall erosivity estimation using the rainfall intensity equation for different climatic regions based on the rainfall erosivity datasets from the United States and Europe.

Further, Panagos *et al*.^11^ developed the Global Rainfall Erosivity Database (GloREDa) using relatively high temporal-resolution rainfall erosivity data (1–60 min) from 63 countries. Liu *et al*.^15^ used a daily erosivity model from limited station sites at a global scale to understand the changes in rainfall erosivity across the globe between 1980 and 2017. More recently, Panagos *et al*.^16^ estimated monthly global rainfall erosivity using more than 45,000 monthly erosivity records from 65 countries. Moreover, in recent decades, efforts^6,10,11,13–16^ have been made to refine the understanding of the global erosivity estimation and reduction in modelling uncertainties. However, the utilization of high-resolution rainfall datasets for estimating rainfall erosivity in global studies has remained limited.

In recent times, noteworthy efforts have been made to update previously developed rainfall erosivity datasets or iso-erodent maps at continental or national levels^17–22^. These initiatives have been complemented by the recent availability of high-resolution rainfall datasets for many countries, providing valuable new insights into spatiotemporal rainfall erosivity patterns^23^. The existing global dataset has been used widely as a primary dataset for comparison and benchmarking in numerous regional^24,25^ and global studies^10,26^. However, the existing global datasets^11,14^ showed regional uncertainty due to the limited availability of gauge stations^18,19,21,27^. Moreover, the existing global rainfall erosivity datasets showed limitations in capturing spatial variation for regions receiving intense rainfall^27^. Furthermore, rainfall erosivity estimated from low-temporal resolution datasets is prone to incorrect estimation of erosivity values^28^. The availability of a high-quality rainfall erosivity dataset is a prerequisite for soil erosion estimation and water resource allocations. Given the limited availability of alternative datasets, the scientific community is compelled to rely on pioneering research based on existing datasets, notwithstanding their acknowledged limitations. Nonetheless, the limitations of existing datasets have become evident, showing the scope of the improvements.

The recent availability of global and regional satellite and reanalysis datasets at the sub-hourly and hourly temporal scales provide an opportunity to estimate rainfall erosivity over a large spatial extent. Many recent studies have been conducted to understand the possible role of rainfall erosivity estimation from the satellite and reanalysis rainfall datasets^10,25,27,29–35^. It is essential to acknowledge that the satellite and reanalysis datasets have inherent uncertainties, which imparts uncertainty in rainfall erosivity estimates^10,24,27,29,34^.

The inherent uncertainty in satellite and reanalysis data-based rainfall erosivity estimates and the uncertainty in the existing global rainfall erosivity datasets limits the reliable use of these products in soil erosion modelling and policy-making. Improving knowledge and reducing uncertainty in global rainfall erosivity datasets with newer high-resolution global datasets is essential for decision-makers and earth-system modellers. For instance, Panagos *et al*.^36^ combined the measured erosivity datasets with General Circulation Models (GCMs), simulating future projections of rainfall erosivity for 2050 and 2070. At the global scale, there is a limited number of rainfall erosivity datasets available, and earth system modellers are seeking new datasets with a fresh perspective. While multisource-based datasets have proven reliable for many hydrological applications^37^, they have been limitedly used in rainfall erosivity estimation. For example, using a non-parametric quantile regression forest, Bhuiyan *et al*.^38^ showed that a multi-source precipitation dataset significantly improved the streamflow simulations. Similarly, Pham *et al*.^39^ illustrated the effectiveness of Artificial Intelligence (AI) models incorporating meteorological parameters in predicting daily rainfall in Vietnam. Additionally, prior studies have explored various merging techniques, including geostatistical interpolation^40,41^ and machine learning-based merging using Support Vector Machine^42,43^, Random Forest^44^ and XGBoost^45^, and Neural Network^46^ to integrate global precipitation products with limited gauge observations. These approaches have effectively addressed the limitations associated with individual satellite and reanalysis datasets. Moreover, the notable success achieved by machine learning models motivates us to extend their application to rainfall erosivity estimation, leveraging multi-source datasets. We employed a Gaussian Process Regression-based machine learning model, which shows promise in enhancing the accuracy and robustness of rainfall erosivity estimation.

**Fig. 1:**
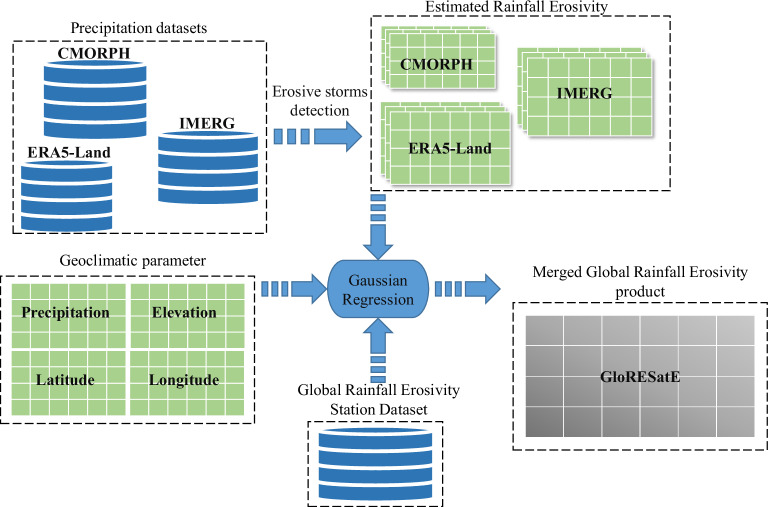
A framework for generating a new global rainfall erosivity dataset.

Therefore, this study aims to present a new global rainfall erosivity dataset generated using a machine learning-based fusion of erosivity estimated from multiple datasets. First, we estimated annual rainfall erosivity from two high-resolution gridded global satellites and one reanalysis precipitation dataset covering the most recent available periods. This process included the estimation of annual rainfall erosivity from satellite-based datasets such as IMERG-Final Run and CMORPH, as well as the reanalysis dataset ERA5-Land. These erosivity estimates and geoclimatic parameters, specifically elevation, latitude, longitude, and mean annual precipitation from the IMERG-Final Run dataset were used to construct a robust regression framework. Within this framework, we integrated a collection of gauge rainfall erosivity data from 6,170 stations across the globe. This amalgamation enabled us to generate a unified global rainfall erosivity dataset, seamlessly merging information from satellite, reanalysis, and gauge records. The workflow of this study is shown in Fig. 1.

## Methods

### Rainfall datasets

The present study utilises two high-resolution gridded global satellites and one reanalysis precipitation dataset for rainfall erosivity estimation. The satellite precipitation datasets used in this study are (a) Climate Prediction Center Morphing Technique (CMORPH) (Version 1) (10.25921/w9va-q159) and (b) Integrated Multi-satellitE Retrievals for GPM (IMERG) (Version 6) (https://disc.gsfc.nasa.gov/datasets) Final Run. The reanalysis dataset used in this study is the Fifth generation of European ReAnalysis (ERA5)- Land (https://cds.climate.copernicus.eu/cdsapp). More detailed information about the datasets can be found in the Supplementary Information. An overview of the datasets used is available in Table 1.

**Table 1:** Overview of satellite and reanalysis precipitation datasets used in this study.

Datasets	Spatial resolution (Approx.)	Spatial coverage	Temporal resolution	Period covered	References
CMORPH (Version 1)	0.07° × 0.07°	60° N-S	30 min	1998–2021	Xie *et al*.^116,117^
IMERG Final Run (Version 6)	0.1° × 0.1°	90° N-S	30 min	2001–2020	Huffman *et al*.^118^
ERA5-Land	0.1° × 0.1°	90° N-S	60 min	2001–2021	Muñoz-Sabater *et al*.^119^

### Global rainfall erosivity station dataset

The rainfall erosivity station dataset was collected by conducting a thorough literature survey of the already published studies. Through an extensive literature survey and survey of regional datasets around the globe, a large number of high-resolution rainfall erosivity station datasets were collected and compiled as a representative database covering different climatic and geographical regions worldwide. This global rainfall erosivity station dataset includes the following datasets:

- A collection of rainfall erosivity datasets of 12 counties across the globe estimated using high-resolution long-term rainfall data. The collection includes rainfall erosivity data from the United States^19,47^, Micronesia^19,47^, China^18^, and Iran^22^. The dataset also consists of the rainfall erosivity literature survey dataset used for rainfall erosivity study over South America^20^ including Argentina^48^, Brazil^49–51^, Chile^52^, Colombia^53,54^, Paraguay^55^, Peru^56^, Uruguay^57^, and Venezuela^58^.
- The rainfall erosivity data collected through the literature survey from 27 countries across the globe. This includes the data of Australia^59–61^, Bangladesh^62^, Brazil^63,64^, Canada^65,66^, Honduras^67^, India^68,69^, Malaysia^70,71^, Mauritius^72^, South Korea^73^, Japan^74,75^, and New Zealand^76^ and the rainfall erosivity literature survey data from 16 countries in Africa used in continental study^29,77^, including Cape Verde^78^ and Canary Islands^79^.
- Data from the recently launched Global Rainfall Erosivity Dataset (GloREDa)^16,80^. From the GloREDa, we used the rainfall erosivity dataset of European countries and other countries’ rainfall erosivity data, including Israel, Japan, Kuwait, Palestine, the Russian Federation, Turkiye, and Yemen. This encompasses rainfall erosivity data from a total of 36 counties.

Detailed information about most of the high-resolution (1–60 min) rainfall erosivity datasets collected and used in this study has been given in Table 2. A high-resolution hourly rainfall dataset from the India Meteorological Department (IMD) (https://dsp.imdpune.gov.in/index.php) was incorporated with temporal coverage from 1969 to 2021. The estimated rainfall erosivity from these 249 stations was further amalgamated into the global rainfall erosivity station dataset, resulting in a comprehensive representation of 296 stations in India.

**Table 2:** Overview of high-resolution long-term mean annual rainfall erosivity dataset used in the study collected from different sources.

Continent	Country/Island	Number of Stations	Temporal Resolution	Start and end year (Approx.)	Author
Africa	Mauritius	4	6 min	2005–2008	Anderson^72^
Australia - Oceania	Australia	159	6 min	1961–2000	Yu, B *et al*.^60,61^
Australia - Oceania	New Zealand	32	10 min	1997–2012	Klik *et al*.^76^
Australia - Oceania	Micronesia, Guam, Hawai	75	15 min	1970–2013	McGehee *et al*.^19^, NCDC^47^
Asia	India	296	1 min, 15 min, 60 min	1994–2015, 1969–2021	Dash *et al*.^68^; Babu *et al*.^69^; This study
Asia	Kuwait, Russian Federation, Israel, Palestinian Territory, Turkiye, Yemen	367	1–60 min	1961–2016	Panagos *et al*.^16,80^
Asia	South Korea	41	5 min	1961–2015	Lee *et al*.^120^; Shin *et al*.^73^
Asia	Malaysia	34	10 min	1999–2008	Leow *et al*.^71^
Asia	Japan	82	10 min, 60 min	1995–2015, 1990–2009	Laceby *et al*.^75^; Santosa *et al*.^74^. Panagos *et al*.^16,80^
Asia	China	173	10 min, 1 min	1998–2002, 1951–2018	Ma *et al*.^121^; Yue *et al*.^18^
Europe	Austria	76	5 min	1995–2015	Johannsen *et al*.^21^
Europe	Czechia	85	10 min	1989–2003	Hanel *et al*.^122^
Europe	Austria, Belgium, Bulgaria, Croatia, Cyprus, Czechia, Denmark, Estonia, France, Germany, Greece, Hungary, Ireland, Italy, Latvia, Liechtenstein, Lithuania, Luxembourg, Netherlands, Poland, Portugal, Romania, Russian Federation, Slovakia, Slovenia, Spain, Sweden, Switzerland, United Kingdom	1804	1–60 min	1980–2022	Panagos *et al*.^16,80,123^; Borrelli *et al*.^111^
North America	United States	2351	15 min	1970–2013	McGehee *et al*.^19^, NCDC^47^
North America	Puerto Rico	22	15 min	1970–2013	McGehee *et al*.^19^, NCDC^47^

**Fig. 2:**
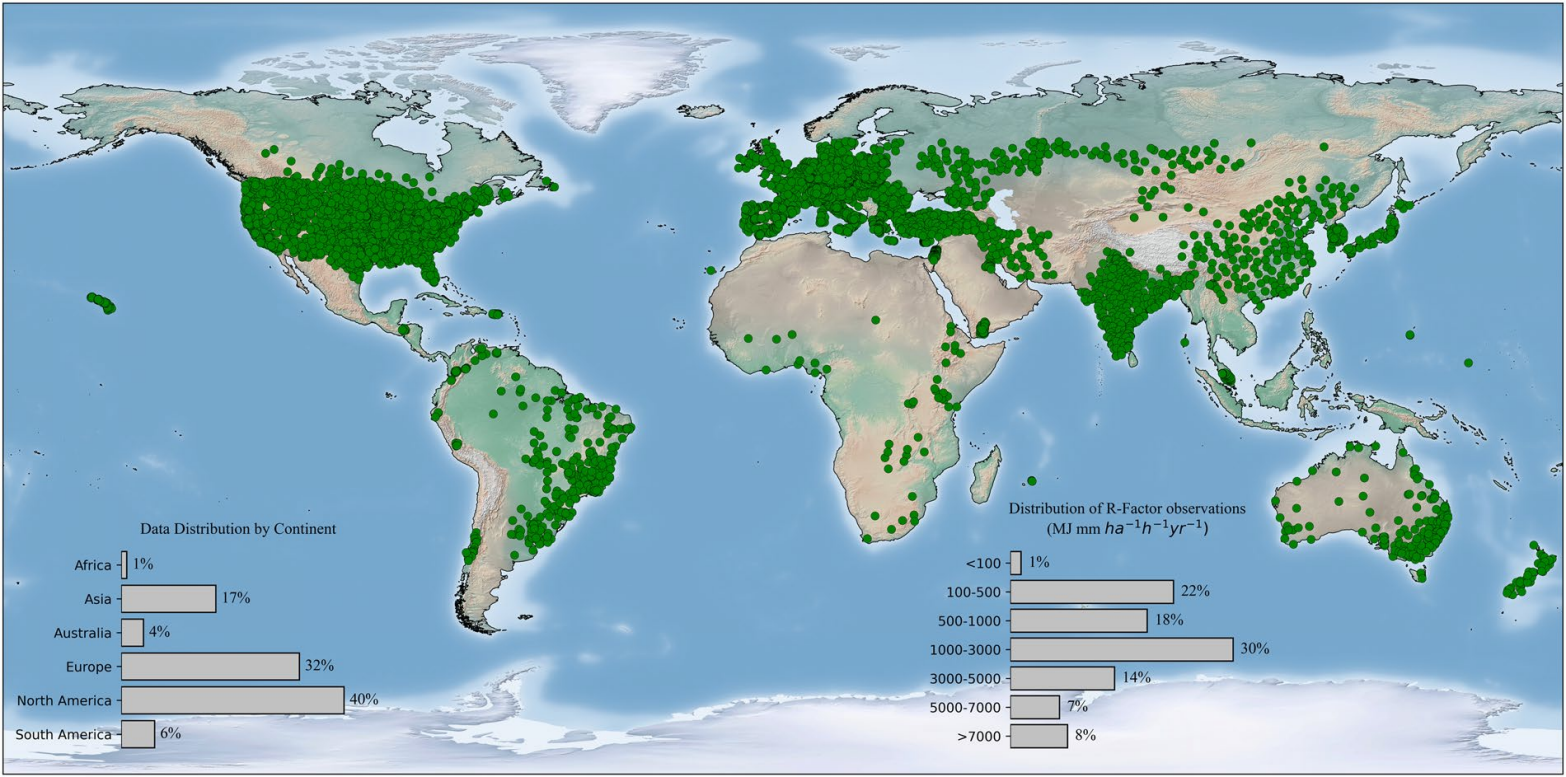
Locations of global rainfall erosivity station (*n* = 6,170) dataset used in this study.

Moreover, the global station dataset used in this study contains representative annual rainfall erosivity values of 6,170 stations covering 72 counties worldwide. The global rainfall erosivity station dataset (Fig. 2) contains ~91% of the stations that use high-resolution rainfall data (≤60 min), and the rest uses the regional rainfall erosivity equation for rainfall erosivity estimation. The number of representative rainfall erosivity stations varies among the continents. North America has the highest number of representative rainfall erosivity stations, with 2,441 stations (~40%), and Africa has the lowest number of rainfall erosivity stations, with 52 stations (~1%). Europe has 1,965 (~32%) stations, the second-highest contributor to the dataset. We have collected 1069 (~17%) and 266 (~4%) stations over Asia and Australia-Oceania, well represented to the erosivity classes. Over South America, 377 (~6%) station data was collected, including data from high erosivity countries Brazil, Colombia, and Venezuela.

The dataset exhibits a comprehensive representation across various climate zones and therefore rainfall erosivity conditions. Approximately 8% of the stations demonstrate very high rainfall erosivity values exceeding 7,000 MJ.mm.ha^−1^.h^−1^.year^−1^, around 7% of total stations have rainfall erosivity values within the 5,000 to 7000 MJ.mm.ha^−1^.h^−1^.year^−1^. Nearly 30% of the stations have rainfall erosivity values ranging between 1,000 to 3,000 MJ.mm.ha^−1^.h^−1^.year^−1^, and approximately 23% register rainfall erosivity values below 500 MJ.mm.ha^−1^.h^−1^.year^−1^.

### Harmonization of different time resolutions in original data

In addressing the variability in the range of available time resolution of data, calibration of erosivity values at different time resolutions becomes necessary. According to GloRESatE statistics, approximately 69.1% of stations provided rainfall data at very high resolution (≤15 min), around 13.3% at intermediate resolution (30 min), and the remaining around 8.8% at a resolution of 60 min. Given this heterogeneity, a calibration process is crucial.

A 30-min time resolution was chosen as an acceptable compromise between the coarse resolution of 60 min and the higher ones (≤15 min). All the rainfall erosivity values obtained from the various temporal resolutions were converted to 30-min rainfall erosivity values using a conversion factor derived from high-resolution rainfall erosivity data from Europe^81^. Numerous studies worldwide have employed similar conversion factors to harmonize different rainfall erosivity datasets^16,82^.

It is worth mentioning that the final version of the global rainfall erosivity station dataset consists of a collection of 30-min rainfall erosivity values harmonized from various temporal resolutions. These calibration factors, developed in the European study, align with range values provided in the literature from studies conducted in China^83^, Italy^84^, India^82^, and the USA^85–87^.

### Rainfall erosivity estimation

The rainfall erosivity was estimated from gridded satellite and reanalysis datasets at temporal resolutions of 30 and 60 min, following the procedure outlined in recent studies^10,27,87^. The rainfall events with a cumulative rainfall of more than 12.7 mm were considered erosive events, and rainfall less than 1.27 mm of more than 6 h was considered dry period^87^. The total kinetic energy of the erosive storm was computed using the rainfall kinetic energy and intensity equation (Eq. 1), a modified version of Brown-Foster relationship^88^. The same equation has been popularly used in recent studies^18,19^ and Revised Soil Loss Equation Version 2 (RUSLE2)^89^. The total kinetic energy (Eq. 2) was estimated, summing the kinetic energy of each time interval. Finally, average annual rainfall erosivity (Eq. 3) was estimated, averaging the total rainfall erosivity of years.

1 $$e=0.29[1-0.72\,\exp (-0.082i)]$$

where *e* is a rainfall kinetic energy per unit depth (MJ.ha^−1^.mm^−1^) for a particular period, and *i* is the rainfall intensity in mm.h^−1^.

The total kinetic energy (*E*) of an erosive event was calculated by summing the multiplication of the rainfall kinetic energy per unit depth (*e*) (MJ.ha^−1^.mm^−1^) and rainfall depth (*θ*) (mm) over the entire period of the erosive storm.

2 $$E={\int }_{0}^{D}\left(e\,\cdot \,\theta \right){dt}$$

where *D* is the total duration of the erosive storm, and *dt* is the time increment of the erosive storm.

The rainfall erosivity was calculated by multiplying the total kinetic energy (*E*) (MJ.ha^−1^) with the maximum 30-minute intensity (*I*_30_) (mm.h^−1^) of the storm. The mean annual rainfall erosivity (*R*) was calculated by summing all storm erosivity values over the years and dividing by the total number of year records.

3 $$R=\frac{1}{n}\mathop{\sum }\limits_{i=1}^{n}\mathop{\sum }\limits_{j=1}^{m}{\left({{EI}}_{30}\right)}_{{ij}}$$

where *R* is the mean annual rainfall erosivity in MJ.mm.ha^−1^.h^−1^.year^−1^, *n* is the number of years, and *m* is the total number of erosive events.

### Merging multi-source datasets

The proposed merged long-term mean annual rainfall erosivity product, Global Rainfall Erosivity from Reanalysis and Satellite Estimates (GloRESatE) is prepared to address uncertainty in satellite and reanalysis erosivity estimates and the limited availability of observed rainfall erosivity dataset globally. We used a machine learning-based regression approach to merge multiple satellite and reanalysis datasets with the station datasets to improve the accuracy of global rainfall estimation. This integration is accomplished by considering geoclimatic covariates. Essentially, this approach aims to identify a relation between the estimated rainfall erosivity from satellite and reanalysis sources, alongside geoclimatic variables, and the global rainfall erosivity station dataset. The covariates used in this study involve

1. Estimated rainfall erosivity: Derived long-term (2001–2020) mean of annual rainfall erosivity estimates from the satellite (IMERG-Final Run and CMORPH) and reanalysis (ERA5-Land) datasets.
2. Geoclimatic parameter: Latitude, longitude of grids, and elevation data from the ~1 km WorldClim^90^ dataset (https://www.worldclim.org/).
3. Climatic parameter: The long-term (2001–2020) mean annual precipitation from the IMERG-Final Run dataset.

Initially, we employed an area conservative regridding technique^91^ to re-grid all datasets into common grids. While various algorithms exist for regridding datasets into common grids, they may not be optimal for rainfall and rainfall erosivity data due to their high intermittency and localised extremes^92^. To address this challenge, we utilised an area-conservative regridding scheme, ensuring the preservation of total values during the process. The regridding from finer to coarser resolution was accomplished using the following equation^92,93^:

4 $${\bar{P}}_{k}=\frac{1}{{A}_{k}}\mathop{\sum }\limits_{n=1}^{N}{\int }_{{A}_{{nk}}}{p}_{n}{dA}$$

where ${\bar{P}}_{k}$ denotes the value of any variable over the designated cell *k* with an area *A*_*k*_. *p*_*n*_ represents the variable value at the source cell *n*, while *N* represents the total number of source cells intersecting within the designated cell. *A*_*nk*_ indicates the intersection area between cells *n* and *k*.

Then, we used Gaussian Process Regression (GPR)^94^ to establish a robust regression framework between derived covariates and gauge rainfall erosivity estimates. The known capability of the machine learning models to capture non-linear relationships between input and output variables, suppressing the constraints of traditional statistical models, is expected to improve global rainfall erosivity estimation. The response variable (*y*) and covariate variable vectors (*x*), the regression function can be written as

5 $$y=f\,(x){\mathscr{+}}{\mathscr{N}}(0,{\sigma }^{2})\,{\rm{with}}\,f\,(x)=K{(x)}^{T}w$$

where *f*(*x*) represents the deterministic part of the model. It combines feature *x* with weights *w*, transformed by the kernel function *K*. The ${\mathscr{N}}(0,{\sigma }^{2})$ represents random noise with mean 0 and variance *σ*^2^.

The Radial Basis Function (RBF) kernel is most commonly used in machine learning; it can model complex relationships between input and output variables. The GPR model was tested for kernel functions, including ‘rbf’ and ‘Matern’ kernels. Among the tested kernels, a ‘rbf’ showed an excellent performance. The Radial Basis Function used in this study can be written as

6 $$k\left({x}_{i}{y}_{j}\right)=\exp \left(-\frac{d{\left({x}_{i}{y}_{j}\right)}^{2}}{2{l}^{2}}\right)$$

where *l* is the length scale parameter (*l* > 0) and *d*(*x*_*i*_*y*_*j*_) is the Euclidian distance between two points *x*_*i*_ and *y*_*j*_.

### Feature importance of predictor variables

In our study, we utilised a machine learning-based Random Forest model to assess the importance of each feature used in our analysis. Random Forest employs a bootstrapping approach where trees are grown in a decorrelated manner. If *B* number of trees are identically distributed with positive pairwise correlation *ρ* and each with variance *σ*^2^, the variance of all trees can be expressed as^95^:

7 $$\rho {\sigma }^{2}+\frac{1-\rho }{B}{\sigma }^{2}$$

As *B* increases, the second term diminishes, indicating that the correlation of bagged tree pairs limits the benefits of averaging. Specifically, when trees are grown, a subset of *m* ≤ *p* input variables is randomly selected as candidates. After B, such trees ${\{T({x;}\,{\theta }_{b})\}}_{1}^{B}$ are grown, the Random Forest predictor is formulated as:

8 $${\hat{f}}_{{rf}}^{B}(x)=\frac{1}{B}\mathop{\sum }\limits_{b=1}^{B}T(x{\rm{;}}{\theta }_{b})$$

where *θ*_*b*_ represents the characteristics of the b-th Random Forest tree in terms of split variables, cutpoints at each node, and terminal node values.

### Optimisation of the model

**Fig. 3:**
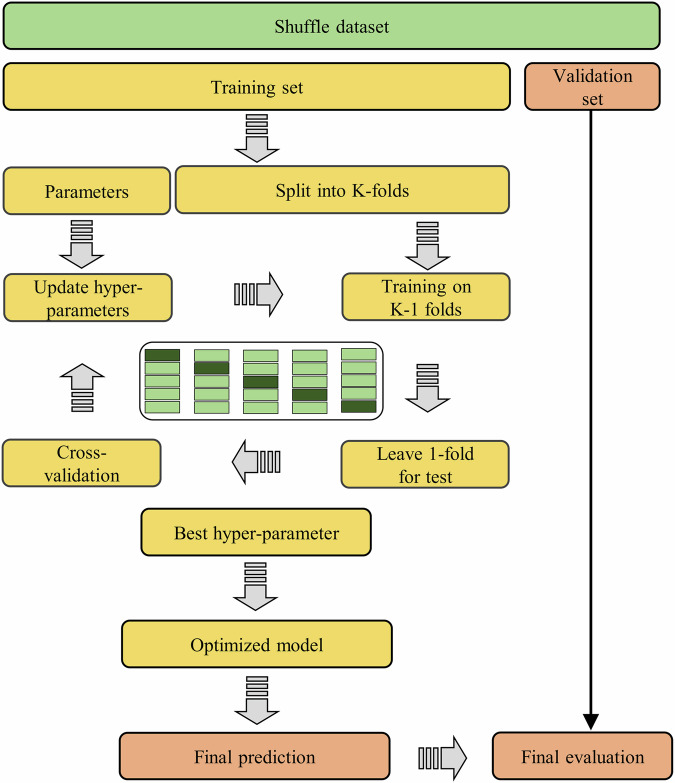
Outline of the methodology used for the model testing.

The process unfolds as follows: First, the entire dataset underwent a randomised splitting process to ensure robust model training and evaluation. Initially, 80% of the entire dataset was allocated for training purposes, with the remaining 20% reserved for validation, as illustrated in Fig. 3. Subsequently, the training dataset was partitioned into K-folds, with K set to 5. This involved dividing the dataset into K equal-sized subsets, utilising K-1 folds for training and the remaining fold for testing. This process was iterated K-times, rotating the testing set each time to ensure comprehensive model assessment.

Moreover, the dataset was categorised into three sets: training, testing, and validation. During the cross-validation procedure, varying sets of the ‘length scale’ and regularisation parameter (‘alpha’) of the GPR model were explored to enhance model performance while minimising errors. This process obtained an optimal value of hyperparameters by maximising the cross-validation score. The Bayesian Optimization Algorithm^96^ (BOA) process effectively optimised the hyperparameters. This algorithm treats the objective function as a black-box function, seeking to maximise the output return value with minimal trials.

Notably, BOA relies on an optimised observation-fitting probability model^97^. An alternative function is created to find the value that minimises the objective function based on the past evaluation results of the objective function. Compared to other methods, BOA selects the parameters based on past evaluation results, saving search time and optimising efficiency^98^. The BOA process includes four primary steps^99^: 1. Defining the hyperparameter search space, 2. Evaluating the test set with a set of hyperparameters, 3. Using the objective function to choose hyperparameter values for the new evaluation, and 4. Reviewing the objective function results, finalizing hyperparameter values, and assessing them on the test set.

### Accuracy evaluation

In this study, we evaluated the dataset performances using four statistical indicators. The indicators include Percentage Error (PE), Pearson’s correlation coefficient (*r*), Nash Sutcliffe Efficiency (NSE), and unbiased Root Mean Square Error (ubRMSE). A more detailed definition of these performance indicators can be found in Supplementary Information and Chicco *et al*.^100^, Moriasi *et al*.^101^, Press *et al*.^102^, Krause *et al*.^103^, and Ma, H. *et al*.^104^.

9 $${PE}=\frac{\left({P}_{i}-{O}_{i}\right)}{{O}_{i}}\times 100$$

10 $$r=\frac{\mathop{\sum }\limits_{i=1}^{n}\left({O}_{i}-\bar{O}\right)\left({P}_{i}-\bar{P}\right)}{\sqrt{\mathop{\sum }\limits_{i=1}^{n}{\left({O}_{i}-\bar{O}\right)}^{2}}\sqrt{\mathop{\sum }\limits_{i=1}^{n}{\left({P}_{i}-\bar{P}\right)}^{2}}}$$

11 $${NSE}=1-\frac{\mathop{\sum }\limits_{i=1}^{n}{\left({O}_{i}-{P}_{i}\right)}^{2}}{\mathop{\sum }\limits_{i=1}^{n}{\left({O}_{i}-\bar{O}\right)}^{2}}$$

12 $${ubRMSE}=\sqrt{E\{{\left[\left({P}_{i}-E\left[{P}_{i}\right]\right)-\left({O}_{i}-E\left[{O}_{i}\right]\right)\right]}^{2}\}}$$

where *O* is the observed values, *P* is the estimated values, $\bar{O}$ is the mean value of observed values, $\bar{P}$ is the mean of estimated values, and *E* represents the mean operator.

## Data Records

The estimated global annual rainfall erosivity for each satellite and reanalysis dataset, at their original spatial resolution, is available on Zenodo^105^ (10.5281/zenodo.11078865). The merged global rainfall erosivity product, estimated from satellite and reanalysis data and incorporating the global rainfall erosivity station dataset, has also been uploaded to the same repository. This product has a spatial resolution of 0.1° × 0.1° and is freely available for download. All the raster datasets are provided with a “.tif” extension, and the estimated rainfall erosivity is in units of MJ.mm.ha^−1^.h^−1^. The long-term mean annual rainfall erosivity values are in MJ.mm.ha^−1^. h^−1^.year^−1^.The details about every file in the repository are as follows:

- “CMORPH.zip”: Contains global rainfall erosivity data estimated from the CMORPH dataset spanning from 1998 to 2021 with a spatial resolution of approx. 0.07° × 0.07°. Each file is named “CMORPH _yyyy.tif”, where “yyyy” represents the year. Each “.tif” file contains 13 bands, with “Band 1” to “Band 12” containing the monthly rainfall erosivity from January to December and “Band 13” containing the annual rainfall erosivity for that year.
- “IMERGFinalRun.zip”: Contains global rainfall erosivity data estimated from the IMERG Final Run dataset spanning from 2001 to 2020 with a spatial resolution of 0.1° × 0.1°. Each file is named “IMERGFinalRun_yyyy.tif”, where “yyyy” represents the year. The Band structure of each “.tif” file is similar to “CMORPH_yyyy.tif” files.
- “ERA5Land.zip”: Contains global rainfall erosivity data estimated from the ERA5 Land dataset spanning from 2001 to 2021 with a spatial resolution of 0.1° × 0.1°. Each file is named “ERA5Land_yyyy.tif”, where “yyyy” represents the year. The Band structure of each “.tif” file is similar to the “CMORPH_yyyy.tif” file.
- “EstimatedMean.zip”: Contains the temporal mean annual rainfall erosivity of all three gridded satellites and the reanalysis dataset. Each file is named “xxxx_mean_yyyy_zzzz.tif”, where “xxxx” is the name of the satellite or reanalysis dataset, “yyyy” is the start year, and “zzzz” is the end year of the mean.
- “GloRESatE.zip”: Contains the merged global long-term mean annual rainfall erosivity product with a spatial resolution of 0.1° × 0.1°, the file named “GloRESatE.tif” and associated uncertainty file named “Uncertainty.tif.”
- “Rainfall Erosivity Data.csv”: Contains the observed global rainfall erosivity station dataset.

## Technical Validation

### Performance of merging

The Gaussian Process Regression (GPR) is a powerful and flexible non-parametric regression technique with the added advantage of probabilistic uncertainty estimation. In this study, we used GPR to merge the multi-source datasets. A comprehensive analysis was conducted on all the parameters utilised for model fitting to ensure robustness. A Random Forest model was employed to assess the importance of each parameter, revealing the potential of overfitting attributed to a single parameter (Supplementary Information). The analysis highlighted the CMORPH R-factor as the most crucial one, given the utilisation of the bias-corrected CMORPH dataset.

Additionally, the IMERG R-factor emerged as another crucial factor for consideration, contributing significantly to model accuracy. The geoclimatic parameters also showed significant importance for the modelling. They were included for the accurate representation, especially in high-altitude regions. The optimised GPR model used in this study demonstrated good performance (Table 3) with a training Nash Sutcliffe Efficiency (NSE) of 0.920, a correlation coefficient of 0.957 and an unbiased Root Mean Square Error (ubRMSE) of 873 MJ.mm.ha^−1^.h^−1^.year^−1^. Through optimisation involving adjustments to the length scale and regularisation parameter (alpha), the model achieved a good cross-validation score of 0.879 from 5-fold with the optimised parameters. Furthermore, the GPR model underwent further evaluation against a separate validation set, revealing a good NSE of 0.876 and an ubRMSE of 1015 MJ.mm.ha^−1^.h^−1^.year^−1^. Overall, the optimised GPR model employed in this study exhibited consistent and robust performance across training-testing and validation sets, highlighting its efficiency and suitability for the merging.

**Table 3:** Selected model parameters and performance during training and validation.

Kernel	Search space	Optimised value	Metrics	Training	Validation
RBF	10^−5^ to 10^5^	Length Scale: 52.47	PE (%)	+7 ± 42	+9 ± 51
			*r*	0.957	0.936
	10^−5^ to 10^5^	Alpha: 0.030	NSE	0.920	0.876
			ubRMSE (MJ.mm.ha^−1^.h^−1^.year^−1^)	873	1015

### Comparison of satellite and reanalysis estimates with observed dataset

**Fig. 4:**
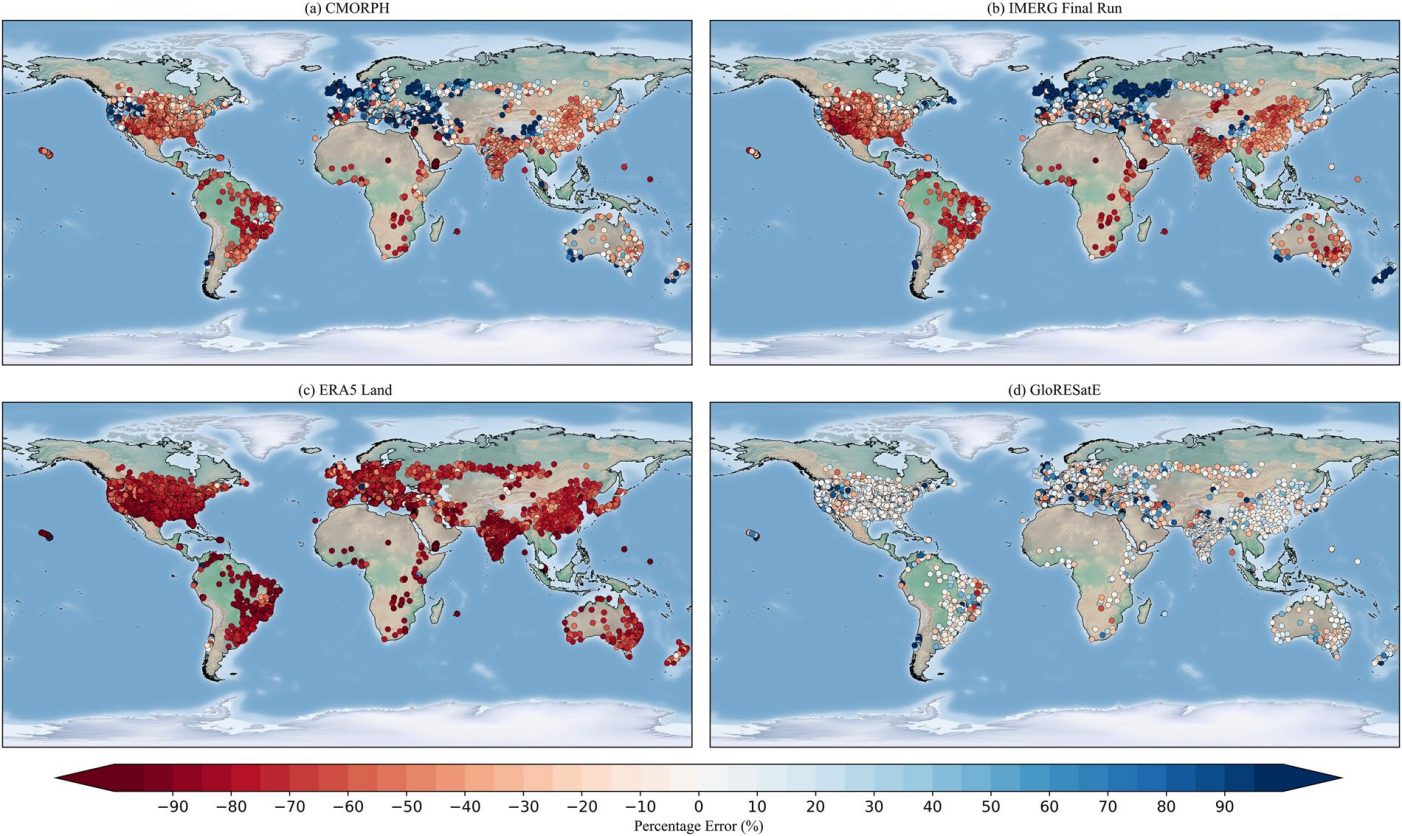
Percentage error in the long-term mean annual rainfall erosivity between global rainfall erosivity station dataset and estimated four rainfall erosivity datasets.

The mean annual rainfall erosivity estimated from satellite and reanalysis datasets from 2001 to 2020 was compared with a global rainfall erosivity station dataset of 6,170 representative stations across 72 countries. Since the ERA5-Land rainfall erosivity was computed from the 60-min precipitation dataset, the extracted rainfall erosivity values were converted to 30-min rainfall erosivity for fair comparison by multiplying the conversion factor^106^ as used for the global rainfall erosivity station dataset. The percentage error with the global rainfall erosivity station dataset has been shown in Fig. 4a–c. At the global scale, the overall mean (±standard deviation) percentage error varies significantly across different datasets, ranging from −70 (±29) % to +10 (±262) % (Table 4). The detailed definitions used for the performance estimation of the datasets are provided in the Supplementary Information. Among the dataset, IMERG-derived rainfall erosivity exhibited a low mean percentage error (−3%) with a high standard deviation (±99%), while ERA5-Land showed a high mean percentage error (−70%). The IMERG-F (Final Run) derived rainfall erosivity displayed a strong positive correlation (*r* = 0.777) with the global rainfall erosivity station dataset at the global scale. Notably, the IMERG-F-derived rainfall erosivity showed the lowest ubRMSE of 2151 MJ.mm.ha^−1^.h^−1^.year^−1^ among all the derived rainfall erosivity datasets. The CMORPH dataset has a similar correlation (*r* = 0.726) with the global rainfall erosivity station dataset. However, the correlation between the reanalysis data-derived rainfall erosivity and the global rainfall erosivity station dataset was low, with a correlation coefficient of less than 0.50. Overall, at the global scale, IMERG-F-derived rainfall erosivity showed better performance than other datasets, exhibiting a stronger correlation, low percentage error, and low unbiased Root Mean Square Error (ubRMSE) compared to the global rainfall erosivity station dataset.

**Table 4:** Evaluation metrics results for the different climatic regions, comparing long-term mean rainfall erosivity estimated from the three rainfall products with the global rainfall erosivity station dataset.

Datasets	Metrics	All-inclusive	Tropical	Arid	Temperate	Cold	Polar
CMORPH	PE (%)	+10 ± 262	−47 ± 38	+37 ± 613	+13 ± 132	+6 ± 97	+92 ± 195
	*r*	0.726	0.553	0.570	0.602	0.775	—
	NSE	0.352	−0.584	0.195	0.167	0.470	—
	ubRMSE (MJ.mm.ha^−1^.h^−1^.year^−1^)	2209	4010	967	2268	845	—
IMERG-Final	PE (%)	−3 ± 99	−50 ± 30	−32 ± 116	+4 ± 111	+11 ± 78	+101 ± 259
	*r*	0.777	0.656	0.637	0.694	0.791	—
	NSE	0.373	−0.597	0.134	0.233	0.538	—
	ubRMSE (MJ.mm.ha^−1^.h^−1^.year^−1^)	2151	3814	927	2119	811	—
ERA5-Land	PE (%)	−70 ± 29	−79 ± 43	−75 ± 23	−70 ± 30	−66 ± 20	−42 ± 77
	*r*	0.356	0.120	0.626	0.407	0.862	—
	NSE	−0.290	−2.307	−0.310	−0.541	−0.237	—
	ubRMSE (MJ.mm.ha^−1^.h^−1^.year^−1^)	2821	5637	1053	2542	993	—

The evaluation results for different climatic conditions are summarised in Table 4. The global climatic regions were identified using the 1-km Koppen-Geiger^107^ climate classification map (Supplementary Information). Due to the limited availability of the rainfall erosivity station data in the polar climate region, only the percentage error (PE) has been estimated. Across tropical climatic regions, the rainfall erosivity estimated from satellite precipitation datasets has a substantial underestimation (mean PE ~ −50%) compared to the global rainfall erosivity station dataset. The reanalysis-derived rainfall erosivity shows an even greater underestimation, exceeding the mean PE by −80%. A low mean percentage error has been observed in the temperate climatic region using the CMORPH (+13 ± 132%) and IMERG-F (+4 ± 111%) dataset, with a notably high standard deviation of percentage error. In the cold climatic conditions, ERA5-Land showed significant underestimation. Across the polar climatic regions, all satellite datasets overestimated rainfall erosivity; however, reanalysis-derived data showed underestimation. Among the satellite-derived rainfall erosivity, IMERG-F exhibited a strong positive correlation (*r* > 0.65) with the global rainfall erosivity station dataset for the tropical climatic regions. Other satellite-derived rainfall erosivity products show a moderate positive correlation with the tropical climatic regions. A similar positive correlation has been observed for almost all the satellite and reanalysis-derived rainfall erosivity products in arid and cold climatic conditions. Furthermore, ERA5-Land-derived rainfall erosivity showed a strong positive correlation of more than 0.80 in cold climatic conditions.

Additionally, we evaluated the performance efficiency of estimated rainfall erosivity from the satellite and reanalysis datasets compared to the global rainfall erosivity station dataset using the Nash-Sutcliffe efficiency (NSE) for different climatic regions. Despite a moderate positive correlation, the results indicated unsatisfactory efficiency (NSE < 0.30) for the tropical climatic regions, suggesting the inefficiency of the satellite-derived rainfall erosivity datasets in accurately estimating rainfall erosivity values for tropical climates. Similar unsatisfactory performances from the satellite and reanalysis-derived rainfall erosivity were observed in temperate and cold climatic conditions.

Furthermore, the ubRMSE for the satellite-derived rainfall erosivity dataset was notably high (~4,000 MJ.mm.ha^−1^.h^−1^.year^−1^) in the tropical climatic regions, with even higher values (~5,500 MJ.mm.ha^−1^.h^−1^.year^−1^) for the reanalysis derived rainfall erosivity. Among the satellite and reanalysis-derived datasets, the IMERG-F-derived rainfall erosivity had the lowest ubRMSE across most climates, showcasing its superior performance. Despite the limitations of satellite and reanalysis datasets at the sub-daily temporal scale, IMERG-F consistently showed more accuracy than other satellite and reanalysis estimates, aligning closely with the outcome of the global review of IMERG dataset^108^. Moreover, it is worth noting that the satellite-derived rainfall erosivity generally performed better than the reanalysis-derived estimates for most climatic conditions. However, over cold climates, reanalysis-derived estimates showed a higher correlation with the observed dataset, possibly due to the limitations of satellite estimates over cold climates^109^. Furthermore, it is important to highlight that none of the satellite and reanalysis datasets consistently exhibited very good performances across all climate regions, underscoring the necessity for significant improvements.

### Comparison of GloRESatE with the existing global product

**Fig. 5:**
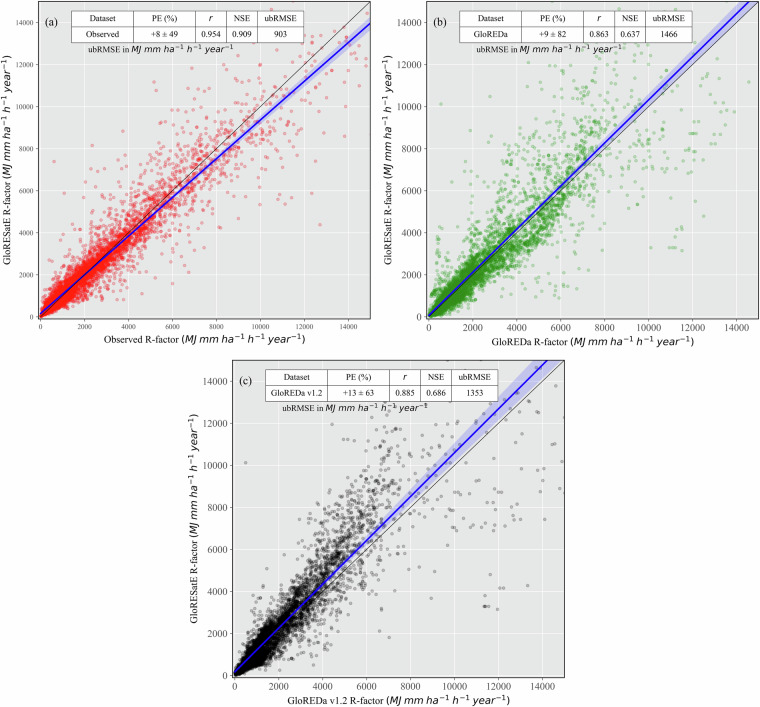
Scatter plot of estimated global rainfall erosivity in this study (GloRESatE^105^) with (**a**) the global rainfall erosivity station dataset, (**b**) the existing global estimates GloREDa (Panagos *et al*.^11^) and (**c**) GloREDa v1.2 (Panagos *et al*.^16^).

The existing 30 arc-seconds (~1 km) global rainfall erosivity map and Global Rainfall Erosivity Database (GloREDa) prepared by Panagos *et al*.^11,16^. showed an incremental improvement over the previous studies. Among the available global datasets, GloREDa has served as a benchmark for many global^10^ and regional studies^24,25,34^ in recent decades. Therefore, the newly developed merged GloRESatE^105^ estimate was compared with the GloREDa versions and global rainfall erosivity station datasets (Fig. 5). The rainfall erosivity values were extracted from the raster datasets (GloREDa and GloRESatE^105^) for the common locations within the global rainfall erosivity station dataset.

The performance was evaluated using Ordinary Least Squares (OLS) regression (Table 5) and evaluation metrics. For a perfect fit, the best-fit line should match the regression line with the black 1:1 line and have an intercept of zero and a regression coefficient close to one. Notably, the regression line between the modelled GloRESatE^105^ rainfall erosivity dataset and the global rainfall erosivity station dataset is close to the 1:1 line and showed an excellent coefficient of determination (R^2^ = 0.910). The regression coefficient is close to one, and the intercept is low. The merged GloRESatE product showed an overall mean percentage error (PE) of only +8 (±49) % and a correlation (*r*) of 0.954. The Nash-Sutcliffe efficiency (NSE) of GloRESatE^105^ modelled data is 0.910, which is much higher than the original satellite and reanalysis-derived rainfall erosivity products. The merged dataset showed a very low ubRMSE value of 903 MJ.mm.ha^−1^.h^−1^.year^−1^, the lowest among other datasets.

**Table 5:** Ordinary Least Squares (OLS) regression statistics of our estimated global rainfall erosivity (GloRESatE^105^) compared to the global rainfall erosivity station dataset and existing global rainfall erosivity datasets (GloREDa^11,16^).

	Global rainfall erosivity station dataset			GloREDa^11^			GloREDa v1.2^16^		
		Std. Error	p-value		Std. Error	p-value		Std. Error	p-value
Intercept	158	14	<0.001	70	26	0.007	171	23	<0.001
Coefficient	0.92	0.004	<0.001	1.02	0.008	<0.001	1.04	0.007	<0.001
R^2^	0.910			0.745			0.784		

When compared to the existing global rainfall erosivity dataset (GloREDa^11^), the GloRESatE^105^ exhibited a coefficient of determination (R^2^) of 0.745, mean percentage error (PE) of +9 (±82) %, and an ubRMSE of 1466 MJ.mm.ha^−1^.h^−1^.year^−1^. Our estimates also showed a good performance with the newly launched GloREDa v1.2, which has a correlation of 0.885 and NSE of 0.686, with a percentage error of only + 13%. Moreover, our dataset correlates more with the new version of GloREDa than the earlier version. The better performance of the developed GloRESatE^105^ rainfall erosivity dataset compared to the original satellite and reanalysis estimates underscores the need to utilise multi-source datasets to improve the performance of the global rainfall erosivity dataset. Furthermore, including more gauge station data in the global rainfall erosivity modelling brings an incremental improvement. Our newly developed global rainfall erosivity product shows a lower percentage error, higher correlation, and enhanced efficiency in capturing the variability in the rainfall erosivity compared to the global rainfall erosivity station dataset and existing Global Rainfall Erosivity Database (GloREDa).

### Comparison of GloRESatE at the continental-scale

We cross-validated the accuracy of the GloRESatE^105^ at the continental scale using statistical metrics with respect to the global rainfall erosivity station dataset. The evaluation results are presented in Table 6. Remarkably, the merged rainfall erosivity product GloRESatE^105^ exhibits good correlations with the global rainfall erosivity station dataset across all six continents. Specifically, over North America, Asia, and Africa, GloRESatE^105^ showed correlation coefficients of 0.970, 0.945, and 0.971, respectively. Moreover, at the continental scale, the GloRESatE showed an excellent correlation value of more than 0.90 for most continents, with a slightly lower correlation of 0.862 observed over Europe and 0.880 over South America. The GloRESatE^105^ showed a low percentage error for all continents, staying within ∓ 10% except for Australia-Oceania, which reached +24%. The excellent Nash-Sutcliffe efficiency (NSE) values were observed over the Africa, Australia-Oceania, Asia, and North American continents. The ubRMSE of GloRESatE remained low over Europe and North America (~500 MJ.mm.ha^−1^.h^−1^.year^−1^); however, it is higher over Africa, Australia-Oceania, Asia, and South America (ubRMSE > 1,000 MJ.mm.ha^−1^.h^−1^.year^−1^). It is important to note that Europe and North America exhibit low mean rainfall erosivity, while Africa, Australia-Oceania, Asia, and South America have significantly higher rainfall erosivity values. The higher ubRMSE values over these continents can be attributed to their high rainfall erosivity values.

**Table 6:** Evaluation metrics results at the continental scale between the newly developed GloRESatE dataset and the global rainfall erosivity station dataset.

Continents	PE (%) (mean ± standard deviation)	*r*	NSE	ubRMSE (MJ.mm.ha^−1^.h^−1^.year^−1^)
Africa	+1 (±26)	0.971	0.932	1433
Asia	+8 (±46)	0.945	0.891	1387
Australia-Oceania	+24 (±50)	0.913	0.822	1553
Europe	+9 (±61)	0.862	0.724	405
North America	+4 (±30)	0.970	0.936	534
South America	+8 (±38)	0.880	0.772	1767

At the continual scale, earlier assessments over Africa^77^ reported a similar NSE of 0.90 and correlation (*r*) of 0.95 using the African Rainfall Erosivity Sub-regional Empirical Downscaling (ARESED) model. Our results over Africa are very close to the ARESED model statistics. Riquetti *et al*.^20^ developed a geographical model to estimate rainfall erosivity over South America with a coefficient of determination (R^2^) of 0.63 and a percentage bias of −1.80%. Our result also showed a similar percentage error over South America with an NSE of 0.772. This also indicates an incremental improvement over South America as this study uses a higher number of stations. The existing rainfall erosivity model for Europe^106^ had a cross-validation score (R^2^) of 0.63 and a fitting score (R^2^) of 0.72 with gauge datasets. Our results also showed a similar performance with an overall score (NSE) of 0.724 over Europe. Using satellite rainfall data and the method proposed by Vrieling *et al*.^29,110^, an existing study over Australia^30^ showed an excellent coefficient of determination (R^2^) of 0.86 with the gauge data. Our result also showed a similar good performance with an NSE of 0.822 over Australia. Moreover, these results collectively show the excellent performance of the merged rainfall erosivity products, GloRESatE^105^, across all continents.

### Comparison of GloRESatE at the regional scale

**Fig. 6:**
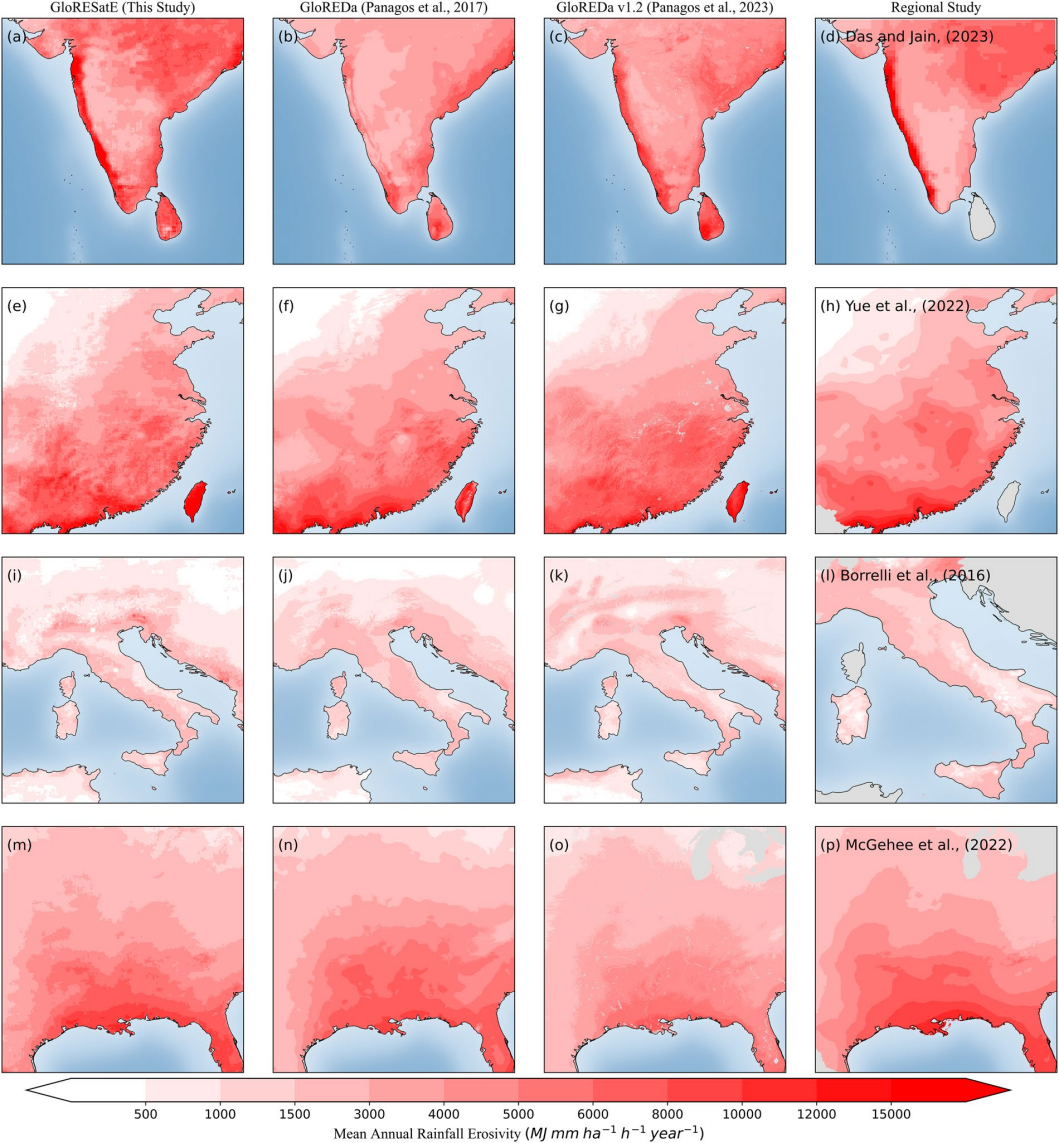
Comparison of spatial maps GloRESatE^105^ datasets (This Study), from GloREDa estimates (Panagos *et al*.^11,16^), and regional datasets for the four different locations (**a**–**d**) part of South Asia, (**e**–**h**) part of Southern Europe, (**i**–**l**) part of East Asia and (**m**–**p**) part of North America.

Further, at the regional level, we conducted a comparative analysis between the GloRESatE^105^ estimates and those from the GloREDa^11,16^, as well as the regional rainfall erosivity datasets from India^82^, Italy^111^, the United States^19^, and China^18^ (Fig. 6). The spatial maps from these datasets for four high erosive regions were plotted side by side. The result shows very similar spatial patterns between GloRESatE and the compared datasets. However, our findings indicated that the coastal regions of India exhibit higher rainfall erosivity compared to earlier estimates of GloREDa. The updated version (GloREDa v1.2) aligns well with our observations. GloRESatE^105^ estimates showed similar spatial patterns over Southern Europe, East Asia and North America when compared to both the versions of GloREDa^11^ estimates and regional studies^18,19^. Although our results indicate relatively higher rainfall erosivity values in these regions compared to GloREDa, this observation aligns well with the findings of regional studies.

In Table 7, we summarise the evaluation results of GloRESatE^105^ with the regional and existing global datasets for four different countries. Our finding revealed a moderate correlation (*r* = 0.608 and 0.688) between the GloREDa estimates and our estimated GloRESatE dataset, but a strong correlation (*r* = 0.786) with the regional study was found over India. Furthermore, compared to the station rainfall erosivity over India, the GloRESatE^105^ shows an overall percentage error of only +10 (±39) % and a good NSE of 0.707. Similarly, over China, the GloRESatE has a +10 ( ± 67) % mean error with the observed station rainfall erosivity dataset. Other statistical measures, such as correlation (*r* = 0.977) and NSE (0.954), also showed excellent GloRESatE performance in China with the observed stations. The GloRESatE dataset showed a PE of +7 (±42) % over the United States with the station erosivity dataset. Over the United States, our estimates revealed an excellent correlation (*r*) of 0.950 and an NSE of 0.900 with the observed stations. Similarly, across the complex topography of Italy, the GloRESatE dataset exhibited a only +9 (±58) % error with a good correlation with the observed dataset. A strong correlation (*r* = 0.853) was observed with GloREDa v1.2 over Italy. Furthermore, the developed GloRESatE^105^ dataset demonstrates a remarkable performance with a significantly lower ubRMSE value. The recent studies in China^18^, United States^19^, India^27^, and Austria^21^ also showed a certain uncertainty in the existing global rainfall erosivity dataset at the regional scale. However, we believe the newly developed rainfall erosivity dataset from the multi-source dataset will help bridge the knowledge gap in the existing global rainfall erosivity dataset.

**Table 7:** Evaluation metrics results at the regional scale between the newly developed GloRESatE dataset and existing datasets.

Region	Dataset	PE (%) (mean ± standard deviation)	*r*	NSE	ubRMSE (MJ.mm.ha^−1^.h^−1^.year^−1^)
India	Global rainfall erosivity station dataset	+10 ± 39	0.846	0.707	2175
	GloREDa^11^	+43 ± 70	0.608	−2.82	3031
	GloREDa v1.2^16^	+26 ± 42	0.688	−0.40	2738
	Das and Jain^82^	+20 ± 36	0.786	0.532	2460
China	Global rainfall erosivity station dataset	+10 ± 67	0.977	0.954	541
	GloREDa^11^	+2 ± 42	0.962	0.925	675
	GloREDa v1.2^16^	−1 ± 32	0.967	0.934	639
	Yue *et al*.^18^	+6 ± 49	0.989	0.967	451
Italy	Global rainfall erosivity station dataset	+9 ± 58	0.770	0.540	671
	GloREDa^11^	+4 ± 31	0.647	0.029	501
	GloREDa v1.2^16^	+8 ± 24	0.853	0.679	345
	Borrelli *et al*.^111^	+20 ± 54	0.702	0.404	520
United States	Global rainfall erosivity station dataset	+7 ± 42	0.950	0.900	687
	GloREDa^11^	+2 ± 44	0.900	0.800	922
	GloREDa v1.2^16^	+37 ± 44	0.930	0.660	808
	McGehee *et al*.^19^	+17 ± 451	0.989	0.967	321

### Summary

Utilising high-resolution precipitation datasets from two satellites (CMORPH and IMERG) and one reanalysis (ERA5-Land), global rainfall erosivity was derived over more than 20 years. Furthermore, a regression-based approach was employed to create a merged long-term mean annual rainfall erosivity product, utilising observed rainfall erosivity data collected from 6,170 stations across 72 countries.

The preliminary analysis revealed significant uncertainty in the long-term mean annual rainfall erosivity estimated from satellite and reanalysis datasets. However, the merged rainfall erosivity product, developed using a machine learning–based Gaussian Process Regression, exhibited significant improvement. Similar merging technique has been used in many studies while merging rainfall datasets from different sources^37–39,42,45,112^. The GPR-based merging approach used in this study demonstrated successful integration of rainfall erosivity from these diverse sources.

Furthermore, in addition to incorporating rainfall or rainfall erosivity products, integrating environmental variables further enhanced the merging process, particularly for locations lacking observed data and high altitudes. Compared to existing datasets, the prepared dataset added rainfall data points globally, leading to good agreement at the regional scale. These results underscore the potential of the dataset prepared using the blending of multi-source datasets. It represents a promising global and regional soil erosion monitoring and water resource management resource.

## Usage Notes

In this study, we present GloRESatE, a global rainfall erosivity dataset that offers mean annual rainfall erosivity data globally, along with estimated modelling uncertainty. The estimated rainfall erosivity from three different gridded satellite and reanalysis datasets for each month and year are also provided for further use in hydrometeorological applications. However, it is imperative to acknowledge and consider the uncertainty associated with satellite and reanalysis datasets for their accurate interpretation and application. Furthermore, these datasets can facilitate the merging of rainfall erosivity derived from satellite and reanalysis estimates at any spatio-temporal scale, thereby reducing the cost of further research and efforts in duplicating work. We recommend using our main product, GloRESatE, instead of estimated rainfall erosivity from satellite and reanalysis datasets for soil erosion, hydrological modelling, water resource management, and climate impact assessment.

Moreover, GloRESatE also holds relevance for policymakers, earth system modellers, and scientists working in fields like agriculture, ecology, and flood management. It presents a harmonised rainfall erosivity product, consolidating data from diverse temporal resolutions into a unified 30-min resolution. While the rainfall erosivity data suits numerous applications directly, users are encouraged to convert it to an alternate resolution if necessary, ensuring compatibility with specific analytical or modeling requirements.

### Limitations and future scopes

The development of GloRESatE involved incorporating a vast number of globally observed station datasets, along with multiple satellite and reanalysis datasets, aimed at enhancing the global rainfall erosivity estimation. However, it is essential to acknowledge certain uncertainties that may have been introduced while compiling global rainfall erosivity station datasets from different sources. These uncertainties can be attributed to several factors: (1) The rainfall erosivity estimates obtained from different temporal resolutions and various estimation methods, including the adaptations of the Universal Soil Loss Equation (USLE)^113^ and its subsequent versions, namely RUSLE^87^ and RUSLE2^89^. (2) The rainfall erosivity estimated from different temporal resolutions was converted to a 30-min rainfall erosivity value using the conversion factor used in the earlier global study^11^. However, this may cause uncertainty at the regional scale^114,115^. (3) The gauge rainfall erosivity dataset estimated from other formulations than USLE^113^ and RUSLE^87^ can have slight deviations, potentially affecting the dataset’s accuracy at the regional scale. Moreover, the fixed interval rainfall dataset has been used for rainfall erosivity estimation. Ideally, a breakpoint dataset should be used in this case^28^; however, due to unavailability, such a dataset was not used. (4) The estimated rainfall erosivity values from breakpoint datasets will be higher than our estimated values^88^. Our results should be verified before benchmarking in any studies. Furthermore, due to the unavailability of the time-series data of observed rainfall erosivity at the global level, the long-term mean annual rainfall erosivity dataset has been prepared in this study. However, a similar assimilation of the multi-source dataset for each year or month can be applied to prepare long-term time series of rainfall erosivity at the regional and global scales.

### Supplementary information

Supplementary information

## Data Availability

Code used in this study is accessible on GitHub (https://github.com/subhankar17th/R-factor.git).
