# Supplementary material for: GloRESatE: A dataset for global rainfall erosivity derived from multi-source data
Source: Sci Data. 2024 Aug 27;11:926. doi: 10.1038/s41597-024-03756-5 (PMC11349900; doi:10.1038/s41597-024-03756-5)
Supplement: Supplementary file 1 — Supplementary information [file 41597_2024_3756_MOESM1_ESM.docx]

**SUPPLEMENTARY INFORMATION**

1. **Rainfall datasets used**
   1. **CMORPH**

The CMORPH climate data record (CMORPH-CDR) is a global high-resolution precipitation dataset that has undergone bias correction and reprocessing using the Climate Prediction Centre (CPC) Morphing Technique (MORPH) developed by the National Oceanic Atmospheric Administration (NOAA). It is available at 30-minute, 60-minute, and daily temporal resolutions, with the 30-minute data having an 8km-by-8km spatial resolution. The dataset covers January 1, 1998, to December 31, 2021, and provides a precipitation rate in millimeters per hour. It was used in the study to determine rainfall erosivity. The National Centres for Environmental Information (NCEI) - NOAA web portal can access the dataset at the link (<https://doi.org/10.25921/w9va-q159>).

- 1. **IMERG**

The Global Precipitation Measurement (GPM) project is a collaboration between the National Aeronautics Space Administration (NASA) and the Japan Aerospace Exploration Agency (JAXA) aimed at providing a global high spatiotemporal precipitation dataset ^1^. The IMERG technique is a unified US algorithm that uses multiple satellite data to estimate precipitation. It employs the Goddard Profiling Algorithm 2017 (GPROF2017) algorithm to calculate precipitation from passive microwave (PMW) sensors, and the data is intercalibrated and adjusted to provide a half-hourly product with a spatial resolution of 0.1°× 0.1°.

The IMERG dataset operates in near real-time, producing 'Early' and 'Late' multi-satellite products and a 'Final' satellite-gauge product prepared once monthly gauge studies are complete. The dataset is accessible in three versions: IMERG-Early Run, available 4 hours after observation. IMERG-Late Run is available 14 hours after observation, and IMERG-Final Run is available ~3.5 months after the observation month. The dataset covers the globe from 90° N to 90° S, with higher reliability within the 60° N to 60° S range due to the lack of infrared estimations over snow and ice-covered areas.

This study utilized the IMERG Final Run datasets with the 30-minute temporal resolution to estimate rainfall erosivity globally from January 1, 2001, to December 31, 2020. The precipitation products include variables 'precipitationcal' and 'precipitationuncal,' and the 'precipitationcal' variable, which includes the correction factor, was used. Since the datasets provide precipitation rates in millimeters per hour for each 30-minute interval, the precipitation intensity was directly used for rainfall erosivity estimation. The dataset can be downloaded from the GES DISC website (<https://disc.gsfc.nasa.gov/datasets>).

- 1. **ERA5-Land**

ERA5-Land is a fifth-generation European Centre for Medium-Range Weather Forecasting (ECMWF) reanalysis dataset providing data for several land variables over several decades with an enhanced spatial resolution of 0.1$^{\circ}\times$ 0.1$^{\circ}$ (~ 9 km) compared to ERA5 ^2^. The dataset has been derived from the HTESSEL land surface model (IFS version Cy45r1) with low meteorological fields from ERA5 ^3^. The dataset is available from January 1, 1950, to the present. The higher spatiotemporal ERA5-Land dataset is useful for all applications, including flood and draught forecasting. The variable' total precipitation' of ERA5-Land with the global coverage from January 1, 2001, to December 31, 2021, was used in this study. The 'total precipitation' of ERA5-Land accumulates hourly rainfall from 00 UTC to the end of the day in the meter of water equivalent. The dataset was converted to total precipitation for each hour in millimeters according to the Copernicus Knowledge Base (CKB) recommendation. The dataset can be downloaded from the Climate Data Store (CDS) (<https://doi.org/10.24381/cds.e2161bac>).

1. **Evaluation matrix**

In this study, we evaluated the model performances using four statistical indicators. The indicators include Percentage Error (PE), Pearson's correlation coefficient ($r)$, Nash Sutcliffe Efficiency (NSE), and unbiased Root Mean Square Error (ubRMSE).

Percentage error (PE) (Eq. 9) quantifies the accuracy of predictions by calculating the difference between observed and predicted values. To comprehensively evaluate the performance of the model datasets. We have employed the mean PE and standard deviation PE as indicators of performance testing. A dataset with a small mean PE and low standard deviation indicates accurate and consistent predictions, ensuring the reliability of the dataset. A negative value indicates underestimation, and a positive value indicates overestimation.

Pearson's Correlation Coefficient (*r*) (Eq. 10) quantifies the linear relationship between observed and predicted values. A value close to 1 indicates a strong positive correlation, while a value close to -1 indicates a strong negative correlation. It helps understand how well the model captures the pattern of the observed data.

Nash-Sutcliffe Efficiency (NSE) (Eq. 11) evaluates the predictive performance of a model by comparing the variability of predicted values with that of observed values. A value of 1 indicates a perfect fit, while values less than 1 suggest varying degrees of model performance.

Unbiased Root Mean Squared Error (ubRMSE) (Eq. 12) measures the average difference between predicted and observed values, considering systematic and random errors. A smaller ubRMSE indicates better model performance and closer agreement between predictions and observations. The ubRMSE has the perfect value of 0; however, getting the ideal value of ubRMSE is next to impossible. Moreover, a lesser ubRMSE indicates a low error, and as the ubRMSE increases, disagreement increases. Also, it is understandable that the ubRMSE value might be higher for high rainfall erosivity regions, especially in tropical climatic regions, than the rainfall erosivity in arid climatic regions.

The ranges of different indicators and their performance definition used in this study have been given in Supplementary Table 1.

Supplementary Table 1. Evaluation metrics used in this study and their performance definitions used in this study

| Metrics | Range | Perfect value | Indicates | Indicates | Indicates | Indicates | Indicates |
| --- | --- | --- | --- | --- | --- | --- | --- |
| PE | -$\infty$ to +$\infty$ | 0 | PE < -50  (Strong underestimation) | -50 < PE < -10  (Moderate underestimation) | -10 < PE < +10  (Satisfactory) | +10 < PE < +50  (Moderate overestimation) | PE > +50  (Strong overestimation) |
| $r$ | -1 to 1 | $\pm$1 | $r$ < -0.75  (Strong negative correlation) | -0.75 < $r$ < -0.25  (Moderate negative correlation) | -0.25 < $r$ < 0.25  (Low correlation) | 0.25 < $r$ < 0.75  (Moderate positive  correlation) | $r$ > 0.75  (Strong positive correlation) |
| NSE | -$\infty$ to 1 | 1 | NSE < 0.3  (Unsatisfactory) | 0.3 < NSE < 0.5  (Low performance) | 0.5 < NSE < 0.7  (Satisfactory) | 0.7 < NSE < 0.9  (Good) | NSE > 0.9  (Excellent) |


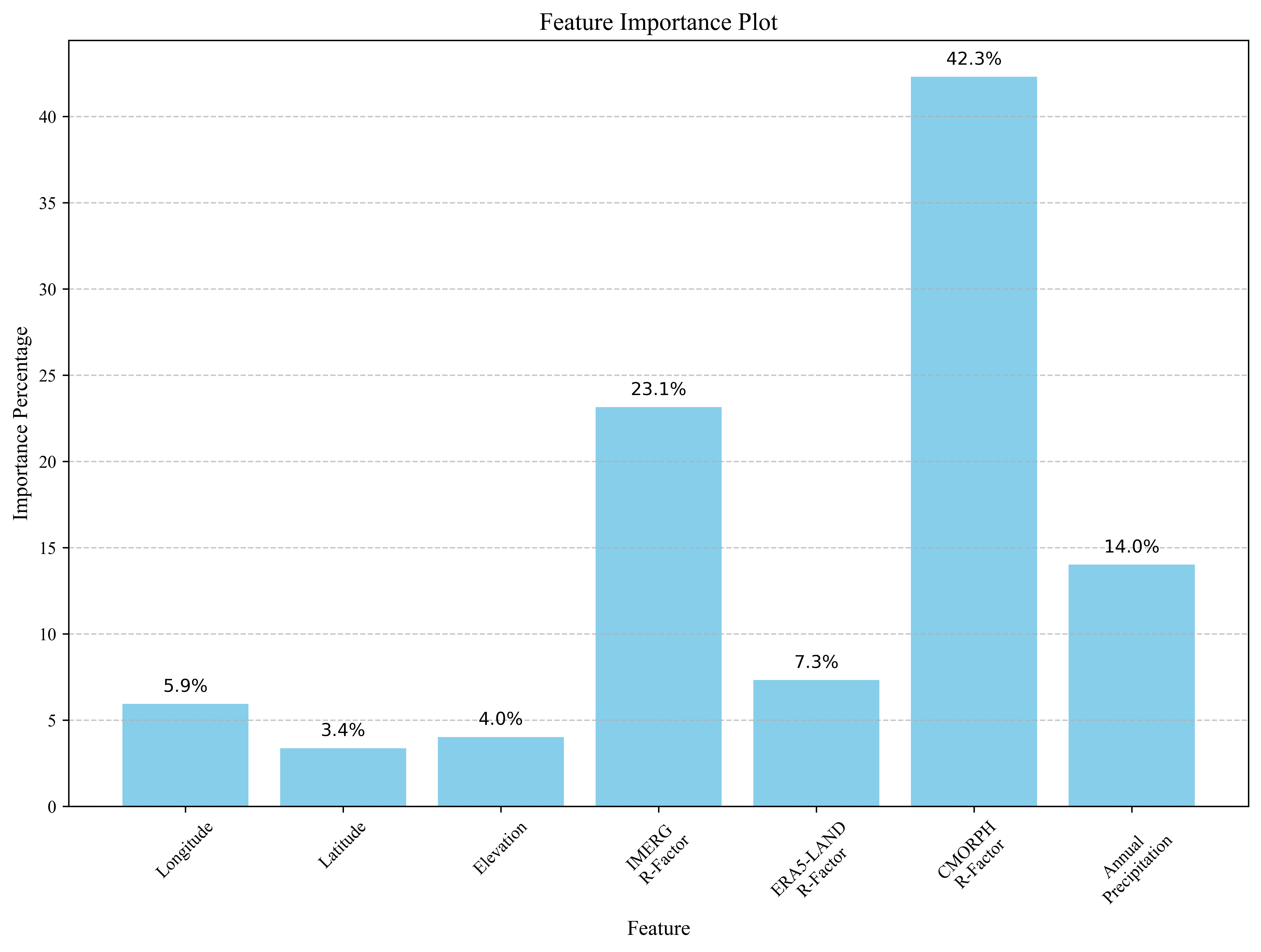


Supplementary Figure 1. Feature importance estimated using a random forest model

1. **Boundaries and maps used**


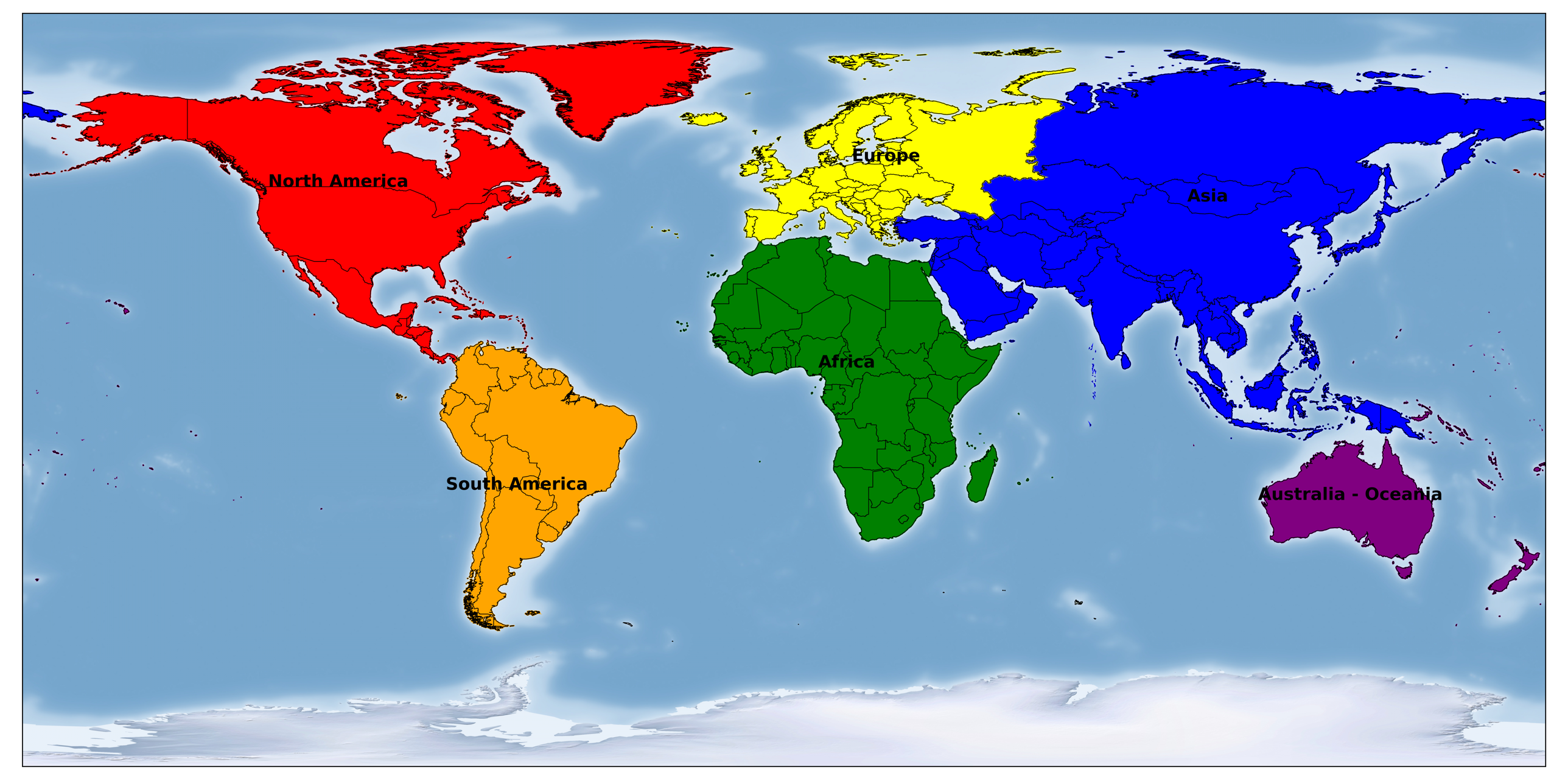


Supplementary Figure 2. The countries and continental borders used in this study. Source: ESRI Word Continents; <https://hub.arcgis.com/datasets/esri::world-continents/explore>, World Countries Generalized; <https://hub.arcgis.com/datasets/esri::world-countries-generalized>.


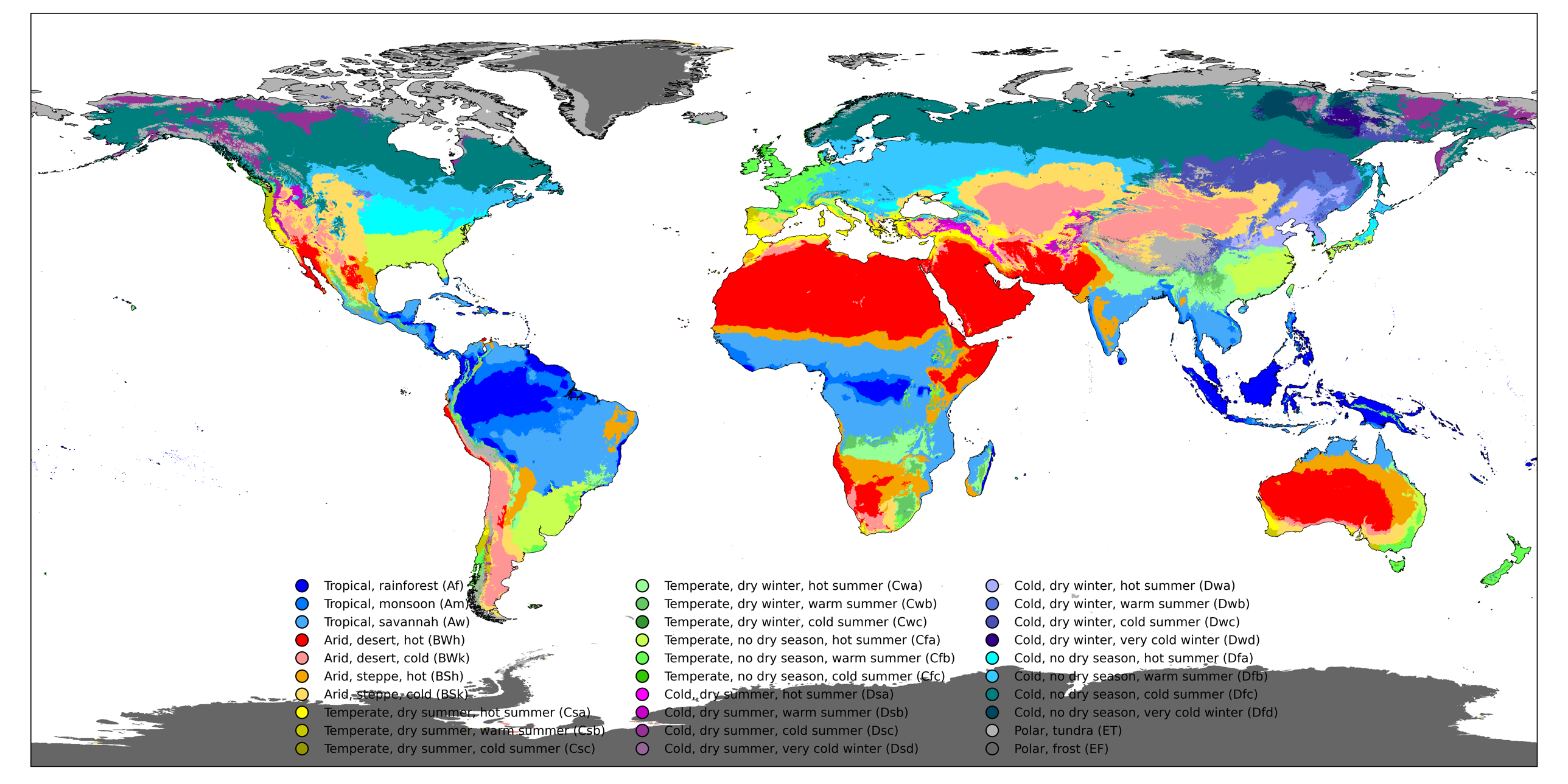


Supplementary Figure 3. This study uses the climate map: Koppen-Geiger climate classification Beck et al. ^4^.

**References:**

1. GJ, H., DT, B., EJ, N. & J, T. Integrated Multi-satellitE Retrievals for GPM (IMERG) Technical Documentation. NASA/GSFC. *Nasa/Gsfc Code* **612**, 83 (2020).

2. Muñoz-Sabater, J. *et al.* ERA5-Land: A state-of-the-art global reanalysis dataset for land applications. *Earth Syst. Sci. Data* **13**, 4349–4383 (2021).

3. Gomis-Cebolla, J., Rattayova, V., Salazar-Galán, S. & Francés, F. Evaluation of ERA5 and ERA5-Land reanalysis precipitation datasets over Spain (1951–2020). *Atmos. Res.* **284**, 106606 (2023).

4. Beck, H. E. *et al.* Present and future köppen-geiger climate classification maps at 1-km resolution. *Sci. Data* **5**, 1–12 (2018).
